# Candidemia in Finland, 1995–1999

**DOI:** 10.3201/eid0908.030069

**Published:** 2003-08

**Authors:** Eira Poikonen, Outi Lyytikäinen, Veli-Jukka Anttila, Petri Ruutu

**Affiliations:** * Peijas Hospital, Vantaa, Finland; † National Public Health Institute, Helsinki, Finland; ‡ Helsinki University Central Hospital, Helsinki, Finland

## Abstract

We analyzed laboratory-based surveillance candidemia data from the National Infectious Disease Register in Finland and reviewed cases of candidemia from one tertiary-care hospital from 1995 to 1999. A total of 479 candidemia cases were reported to the Register. The annual incidence rose from 1.7 per 100,000 population in 1995 to 2.2 in 1999. Species other than *Candida albicans* accounted for 30% of cases without change in the proportion. A total of 79 cases of candidemia were identified at the hospital; the rate varied from 0.03 to 0.05 per 1,000 patient-days by year. Predisposing factors included indwelling catheters (81%), gastrointestinal surgery (27%), hematologic malignancy (25%), other types of surgery (21%), and solid malignancies (20%). Crude 7-day and 30-day case-fatality ratios were 15% and 35%, respectively. The rate of candidemia increased in Finland but is still substantially lower than in the United States. No shift to non–*C. albicans* species could be detected.

A number of reports indicate a substantial increase in candida infections in the United States during the last 2 decades, including a consequent rise in related deaths and prolonged hospitalization (*1*–*5*). *Candida* sp. have been shown to be the fourth most common group of organisms causing nosocomial bloodstream infections (BSI) in the United States (*6*–*9*). Reports also suggest an increase in candidemia in Europe and Australia (*10*–*12*). Factors contributing to this trend are a growing population of immunocompromised patients and the use of new, aggressive, and invasive therapeutic strategies (*6*,*13*). Although most candidemia cases are due to *Candida albicans*, infections caused by non–*C. albicans* species have become more common (*8*,*9*,*12*,*14*–*18*).

For the most part, the epidemiology of candidemia has been studied in selected hospitals, which may not be representative of all hospitals serving a population (*11*,*12*,*19*–*24*). Few population-based studies identifying trends in the incidence of candidemia over time have been published, and the absolute numbers for age- and sex-specific incidence rates have rarely been reported (*18*,*25*–*28*).

We evaluated trends in the incidence of BSIs caused by C*andida* spp. in Finland from 1995 to 1999, using data on BSIs from laboratory-based surveillance introduced in 1995. We also reviewed the characteristics of candidemia cases that occurred in the largest tertiary-care hospital in Finland during the same period.

## Methods

### Surveillance and Population

Finland (population 5.2 million) has five tertiary-care hospitals, with well-defined catchment populations of 0.71 to 1.66 million. Since 1995, all clinical microbiology laboratories in Finland have reported all bacterial and fungal isolations from blood, including *Candida* spp., to the National Infectious Diseases Register. Detection and species determination of *Candida* isolates are performed in the notifying laboratories according to standard protocols in use in each laboratory. Data collected with each notification include the date of isolation, date of birth, sex, type of specimen, and place of treatment.

A case of candidemia was defined as a patient with at least one blood culture positive for *Candida* species. Notifications of the same species of *Candida* within 3 months after the first diagnostic sample in the same patient were defined as one case. Isolations of the same species beyond this time period in the same patient were defined as separate cases.

### Tertiary-Care Hospital

Helsinki University Central Hospital (HUCH) is a tertiary-care hospital with 1,600 beds that serves a population of 1.66 million living in the Helsinki area in southern Finland. In some specialties, such as bone marrow and solid organ transplantation, HUCH provides national service. All patients with at least one blood culture positive for *Candida* from January 1995 to December 1999 were retrospectively identified from the microbiology laboratory logbooks of HUCH’s department of bacteriology. Nosocomial versus community acquisition was defined according to proposed standard criteria (*29*). The following data were abstracted from patient charts: type of specialty, underlying conditions, central venous catheters and bladder catheters in place, cultures taken from these catheters, and the outcome of the illness. Immunosuppressive status was defined as cytotoxic therapy or total body irradiation <3 months before onset of candidemia or systemic cortisone (>40 mg per day at onset of cortisone treatment) <1 month before onset of candidemia. The annual numbers of patient days and discharges were acquired from the hospital administration. No guidelines for systematic antifungal prophylaxis for any patient groups were in use in HUCH during the study period.

### Incidence Rates and Statistical Analysis

Data from the Finland National Population Registry from 1995 to 1999 were used as denominators to calculate age- and sex-specific incidence rates. Average annual incidences during the surveillance period were calculated by using the total number of cases and population during 1995 to 1999. To evaluate secular trends, rates of candidemia in different age and sex groups were calculated for each 6-month period from January 1995 to December 1999. If changes were detected, Poisson regression model was used to assess whether the observed changes in the rates were statistically significant.

Data were analyzed by using Epi Info version 6.04 (available from: URL: http://www.cdc.gov/epiinfo/ei6.htm) and SPSS for Windows version 11 (Chicago, IL). Categorical variables were analyzed with the chi-square test, Yates’s correction, or Fisher exact test, as appropriate. Continuous variables were analyzed by Student t test or the Mann-Whitney U test, depending on the sample distribution.

## Results

A total of 479 candidemia cases were reported to the National Infectious Diseases Register from 1995 to 1999. The median age of the patients was 59 years of age (range 0–89 years); 266 (60%) were males. The average annual incidence of candidemia was 1.9 per 100,000 population and varied from 1.3 to 2.2 in the five tertiary-care hospital catchment areas. The incidence increased from 1.7 per 100,000 population in 1995 to 2.2 in 1999. The average annual incidence of candidemia was highest in infants <1 year of age and lowest in patients 1–15 years of age (Table 1); infants <1 year of age accounted for only 6% of all candidemia cases. In all age groups, the incidence was higher in males than in females. In males 16–65 years of age, the incidence rose significantly, from 1.0 per 100,000 population in 1995 to 2.4 per 100,000 population in 1999 (p<0.05 by Poisson regression); by 1999, the incidence rate for males was three times the rate in females. No trends were identified in other age and sex groups. The highest annual incidence (24.4/100,000 population) occurred in 1999 in infants <1 year of age, which was primarily caused by *C. albicans* (11 cases, 5 of which occurred in one tertiary-care hospital).

**Table 1:** Annual incidence of candidemia by sex and age group, Finland, 1995–1999

Characteristics		Rate^a^															
		1995			1996				1997	1998				1999	1995–1999		
Males (y)																	
<1		12.5			10.0		3.3					6.9	27.3			11.9 (18)	
1–15		1.2			0.4		0.6					0	0.2			0.5 (12)	
16–65		1.0			1.9		1.9					2.1	2.4			1.8 (159)	
>65		7.7			6.3		6.1					9.4	7.7			7.4 (97)	
All		1.9 (46)			2.1 (53)		2.1 (53)					2.5 (63)	2.8 (71)			2.3 (286)	
Females (y)																	
<1	6.5			3.4				0				3.6	21.3				6.9 (10)
1–15	0.6			0.4				0.6				0.2	0.6				0.5 (12)
16–65	1.0			0.8				1.1				1.2	0.8				1.0 (84)
>65	4.2			4.3				3.4				3.6	4.2				3.9 (87)
All	1.5 (39)			1.4 (36)				1.4 (37)				1.5 (39)	1.6 (42)				1.5 (193)
All (y)																	
<1	9.6		6.6			1.7					5.3		24.4				9.4 (28)	
1–15	1.0		0.5			0.6					0.1		0.4				0.5 (24)	
16–65	1.0		1.4			1.5					1.7		1.6				1.4 (243)	
>65	5.4		5.0			4.4					5.7		5.6				5.2 (184)	
All	1.7 (85)		1.7 (89)			1.8 (90)					2.0 (102)		2.2 (113)				1.9 (479)	

^a^Cases per 100,000 population (no. of cases).

The most frequent *Candida* sp. encountered was *C. albicans*, which caused 335 (70%) cases (Table 2). The most common non–*C. albicans* species found was *C. glabrata*, followed by *C. krusei*, *C. parapsilosis*, and *C. tropicalis*. The other species reported were *C. pelliculosa* (five cases) and *C. rugosa* (one case). In 14 (3%) cases, the species were not specified. The proportion of non–*C. albicans* species did not increase during the study period.

**Table 2:** Distribution of *Candida* spp. causing bloodstream infections, Finland, 1995–1999^a^

*Candida* species	1995		1996		1997		1998		1999		1995–1999	
*C. albicans*	67	(57)	75	(67)	73	(66)	61	(62)	73	(83)	70	(335)
Non-*C. albicans*	33	(28)	25	(22)	27	(24)	39	(40)	27	(30)	30	(144)
*C. glabrata*	14	(12)	3	(3)	8	(7)	8	(8)	10	(11)	9	(41)
*C. krusei*	5	(4)	12	(10)	4	(4)	15	(16)	5	(6)	8	(40)
*C. parapsilosis*	11	(9)	1	(1)	6	(5)	6	(6)	5	(6)	5	(27)
*C. tropicalis*	1	(1)	3	(3)	1	(1)	5	(5)	3	(3)	3	(13)
Other	2	(2)	6	(5)	8	(7)	5	(5)	4	(4)	5	(23)

^a^Percent (no.).

In the study hospital, we identified a total of 86 candidemia cases. All but one case were determined to be nosocomial. Four of the 85 nosocomial candidemia case-patients were associated with predisposing treatment in other hospitals, and 2 additional cases-patients, for whom the clinical information was not available, were excluded, leaving 79 patients with cases of nosocomial candidemia for detailed analysis. The median duration of hospital stay before onset of candidemia was 19 days (range 0–177 days). The median age of the patients was 56 years (range <89 years); 45 (57%) of the patients were male.

The average annual incidence of candidemia at HUCH was 0.17 per 1,000 discharges (range by year 0.12–0.21) and 0.04 per 1,000 patient days (range by year 0.03–0.05). Male patients accounted for 70% of cases <1 year of age and 66% of those 16–65 years of age, whereas 64% of the cases >65 years of age were women. We found no increase in the annual number of cases in men 16–65 years of age, in contrast to the increasing rate identified in the national population-based analysis.

At the onset of candidemia, all cases in the study hospital had at least one predisposing factor. Of 79 cases, 19% were leukopenic (leukocytes <1 x 10E9/L), and 14% were neutropenic (neutrophils <0.5 x 10E9/L); 44% were immunocompromised. Gastrointestinal surgery was the most common underlying condition, followed by hematologic malignancy, other surgery, and solid malignancies (Table 3). Solid organ transplantation preceded onset of candidemia in three cases and bone marrow transplantation in five. None of the case-patients had HIV infection. At the onset of candidemia, 18 cases (23%) were treated in intensive-care units (ICU). Nine (50%) of 18 ICU cases were treated in neonatal ICUs. These 9 neonatal ICU case-patients were preterm and constituted 90% of infants <1 year of age; mean gestational age was 28 weeks (range 23–39), and mean birth weight 1,129 g (range 450–3,340 g). Before onset of candidemia, 64 (81%) of case-patients had a central venous catheter and 33 (42%) had a bladder catheter in place. Central venous catheter tip culture was positive for *Candida* in 28 (44%) of the 64 cases, and urine culture was positive for *Candida* in 7 (21%) of the 33 patients with a bladder catheter. Biopsy-proven deep *Candida* infection was detected in 9 cases (11%); all had central venous catheters, and 8 (89%) were operated before onset of candidemia.

**Table 3:** Predisposing factors among 79 patients with nosocomial candidemia, Helsinki University Central Hospital, 1995–1999^a^

Predisposing factor	No. (%)	
Central venous catheter	64	(81)
Urinary catheter	33	(42)
Gastrointestinal surgery^b^	22	(27)
Hematologic malignancy	20	(25)
Other surgery^a^	17	(21)
Solid malignancy	16	(20)
Diabetes	14	(18)
Newborn status	9	(11)
Organ transplantation	8	(10)
Severe trauma	2	(3)

^a^One patient may have several predisposing factors.

^b^Surgery during the same hospital period as candidemia, or within 1 month before the first blood culture.

The most common *Candida* species at HUCH was *C. albicans* (55 cases, 70%), consistent with the overall national figure (Table 2). For non–*C.* *albicans* species, the proportion varied by year from 17% in 1997 to 37% in 1999. In contrast to the national data, *C. parapsilosis* was as common as *C. glabrata* at the study hospital (six cases each).

Among the 79 patients with candidemia at HUCH, 12 (15%) died within 1 week after onset and 28 (35%) within 1 month. Of those who died, one patient had had preceding treatment in ICU. The patients who died were significantly older (median age 51 vs. 60 years of age, p<0.05) and were more likely to have hematologic malignancies (60% vs. 27%, p<0.05).

## Discussion

Our nationwide population-based study shows that the incidence of candidemia in Finland is relatively low. However, we found a consistent year-to-year increase, mainly attributable to an increase in the incidence among men 16–65 years of age. No shift towards non–*C. albicans* species was observed.

We analyzed laboratory-based surveillance data on BSIs caused by *Candida* spp. from nationwide surveillance; therefore, our estimates are representative of the whole population. The rate we found is one third to one fourth of the rates reported from the United States (6.0–8.0/100,000 population) (*18*,*25*,*26*). Two of the U.S. reports included selected urban areas, and the third one was based on sentinel surveillance implemented in selected laboratories in Iowa (which may not be representative of the general U.S. population). A nationwide study from Iceland also documented an increase in the incidence of candidemia from 1.4 per 100,000 population in 1980 to 1984 to 4.9 in 1995 to 1999 (*27*). The 1995–1999 rate in Iceland was more than twice as high as the rate we observed in Finland during the same period. Another nationwide study from Norway reporting the annual numbers of fungemia cases did not identify any change in the period 1991–1996 (*28*). This study also included non-*Candida* yeasts. We did not identify any shift towards non–*C. albicans* species, in contrast to several reports from the United States, Australia, and Europe (*12*,*14*,*16*,*18*) but in accordance with the nationwide studies from Iceland and Norway (*27*,*28*).

We observed the highest age-specific incidence rate in infants <1 year of age. This rate is, however, substantially lower than the rates reported from the Atlanta and San Francisco Bay areas in 1992 and 1993 in the same age group (9.4 vs. 75/100,000 population) (*18*). We found a substantial increase in candidemia cases in men 16–65 years of age. The reason for this increase remains unknown; the detailed analysis in the largest tertiary-care hospital in Finland during the same period showed no increase or major change of characteristics in this demographic subgroup. The Icelandic study showed that the incidence was highest in the elderly and that the increase occurred most in the youngest age group (*27*). The male dominance we observed is similar to that found in previous reports (*18*,*25*,*26*,*28*).

At the largest tertiary-care hospital in Finland, the average annual incidence of candidemia per 1,000 patient days was considerably less than that in the United States (0.04 vs. 2.15), as was the rate per 1,000 discharges (0.17 vs. 0.6) (*2*,*30*). Incidences similar to the current study have been reported from European (*11*,*22*–*24*,*28*) and Australian tertiary-care centers (*12*). We observed that non-nosocomial candidemia was very rare in this tertiary-care hospital (1/84 cases), which is in strong contrast to reports from the United States, where one fifth of candidemias developed in patients before or on admission to hospital (*18*).

Differences in candidemia rates between countries may also be attributable to differences in the representativeness of the study population, the prevalence of HIV infection in the study population, and variations in patterns of healthcare delivery and clinical practices, including the frequency of using blood cultures in diagnostics. The differences may also be explained by differences in antibiotic use patterns and resistance situation (*28*). A previous study from Finland on nosocomial BSIs showed that *Candida* spp. represented only 4% of all findings and the prevalence of antibiotic resistance among bacterial findings was lower than in the United States (*31*).

The role of fluconazole prophylaxis is well established in neutropenic patients; however, among patients without neutropenia, such as surgical ICU patients, this role is less definitive (*32*–*34*). Clinical practices in prophylaxis policies may vary a great deal between institutions and countries. While the prophylaxis effectively reduces the incidence of infections caused by fluconazole-sensitive species, the drug has an impact on the distribution of causative *Candida* species (*11*,*15*,*32*–*35*). Although national data on fluconazole usage are available from Iceland and Norway, they are not comparable (*27*,*28*).

Our study confirms the importance of surgery, cancer, and hematologic malignancies as factors contributing to nosocomial candidemia. Only 23% of our candidemia patients were being treated in ICUs. Approximately twice the proportion of patients with candidemia who had had preceding treatment in ICU was reported from Italy (*21*,*24*), but a similar proportion to ours was reported from France (*23*). Our patient population included no HIV patients, which reflects the low prevalence of HIV infection in Finland (10–16/100,000 population in 1995–1999) (*36*). This low prevalence may substantially contribute to both the lower overall incidence of candidemia and the low proportion of non-nosocomial *Candida* infection in Finland, since in the United States the proportion of candidemia cases with HIV infection to all candidemia cases varied from 10% to 15% (*18*,*25*). The contribution of HIV infection as a predisposing risk factor for candidemia is further emphasized by a report from Italy, where *Candida* spp. was the third most common cause of nosocomial BSI in HIV patients (*37*). In France, among cases of nosocomial candidemia, 13% of patients were reported to have HIV infection during 1990–1995 in one institution (*38*).

The high case-fatality ratio we observed in the older age groups and in patients with a hematologic malignancy reflects the combination of serious underlying diseases and the intensity of treatments modifying host defense that leads to candidemia. In our study, the overall case-fatality ratio of 15% during 1 week and 35% within 1 month after the onset of candidemia are similar to ratios reported from Europe (*21*,*24*) and the United States (*39*), with case-fatality ratios ranging from 35% to 39%, respectively, within 1 month after onset of candidemia.

The results of this study demonstrate a low but consistently increasing incidence of candidemia in Finland. The high case-fatality ratio emphasizes the need for continuous surveillance to identify changes in predisposing factors for optimizing prevention policies, including the use of antifungal prophylaxis.
